# Rituximab for refractory minimal change disease: Long-term outcomes in the remission maintenance phase

**DOI:** 10.1097/MD.0000000000046798

**Published:** 2025-12-26

**Authors:** Li Tian, Lingling Xing, Liying Wen, Ranjie Fu, Xuzhi Liang, Shaomei Li

**Affiliations:** a Department of Nephrology, The Second Hospital of Hebei Medical University, Shijiazhuang, China.

**Keywords:** corticosteroids, minimal change disease (MCD), refractory nephrotic syndrome, relapse, rituximab (RTX)

## Abstract

Minimal change disease (MCD) has a high recurrence rate, with refractory MCD frequently requiring long-term corticosteroid therapy or combination regimens with other immunosuppressive agents. Rituximab (RTX) has been confirmed to be an effective treatment for refractory MCD. However, its role in remission maintenance remains uncertain. This study aimed to assess RTX dosing strategies, infusion intervals, and relapse predictors during the remission maintenance phase of refractory MCD. We conducted a retrospective analysis of 14 patients with refractory MCD who had achieved complete remission with corticosteroids or conventional immunosuppressants and subsequently received RTX during the maintenance phase. The mean follow-up period was 31.1 ± 9.6 months. RTX was administered intravenously at a dose of 375 mg/m^2^ body surface area or 1000 mg every infusion. Based on their laboratory results, RTX therapy was administered in the patients intermittently. Patients who experienced recurrence were withdrawn from the study for further observation and management. Patients were treated with RTX at 375 mg/m^2^ body surface area or 1000 mg with each infusion in the remission maintenance phase. The mean intervals from the first to second and from the second to third infusions were 7.9 ± 2.1 months and 11.8 ± 2.8 months, respectively. Eight patients remained in remission and 6 patients experienced a relapse. Steroids or immunosuppressants were discontinued in 12 patients, while a dose reduction was achieved in 2 others. The relapse frequency significantly decreased from 1.87 (0.43–2.69) to 0 (0.00–1.33; *P <* .05). Recurrence was negatively associated with additional annual RTX therapy (*P* < .05). Serious side effects were not found. RTX is associated with reduced relapse frequency and earlier discontinuation of corticosteroids and immunosuppressants in refractory MCD patients during remission maintenance. Additional annual RTX administration represents an effective strategy for decreasing disease recurrence. Our findings suggest potential benefit of early administration. While findings are promising, larger controlled studies are necessary to establish optimal dosing intervals and long-term efficacy.

## 1. Introduction

Minimal change disease (MCD) affects approximately 10% to 15% of adult patients with idiopathic nephrotic syndrome (NS). Glucocorticoids are recommended as first-line therapies for MCD according to kidney disease: improving global outcomes.^[1]^ The response rate is reported to be 75%. Cyclosporine A, cyclophosphamide, mycophenolate mofetil, azathioprine, tacrolimus and levamisole are used as second-line therapeutic approaches.^[2]^ However, MCD has a high recurrence rate. Corticosteroids and the aforementioned immunosuppressive agents can be employed to manage refractory MCD, but their use is often met with significant apprehension due to the spectrum of potential adverse effects. Therefore, a therapy with a higher level of patient satisfaction is desirable.

Rituximab (RTX) is a chimeric murine/human monoclonal immunoglobulin antibody that binds to CD20-positive lymphocytes and inhibits their proliferation and differentiation. It is used to treat non-Hodgkin lymphoma, rheumatoid arthritis and antineutrophil cytoplasmic antibody-associated vasculitis. RTX has been proven to be a safe and effective therapy for MCD,^[3–7]^ especially for frequently relapsing or steroid-dependent MCD. However, its role in remission maintenance remains uncertain. This study aimed to assess RTX dosing strategies, infusion intervals, and relapse predictors during the remission maintenance phase of refractory MCD.

## 2. Study design and population

In this retrospective study, we collected fourteen MCD patients (Age > 14 years) diagnosed with biopsy confirmation. These patients who had achieved complete remission (CR) with corticosteroids or conventional immunosuppressants and subsequently received RTX during the maintenance phase at The Second Hospital of Hebei Medical University between May 2021 and May 2025. Inclusion criteria was no previous history of RTX. Exclusion criteria were: known associated systemic disease, positive serology for hepatitis B and C, HIV and antinuclear antibodies.

Given that this study was a retrospective cohort study and all clinical information was de-identified, the requirement for informed consent was waived. This study was approved by the Ethics Committee of the Second Hospital of Hebei Medical University (2024-R399). The study was conducted in accordance with the principles of the Declaration of Helsinki.

## 3. Clinical characteristics

We retrieved the following clinical and laboratory data: baseline characteristics (age at disease diagnosis, age at RTX start, sex, comorbidities, systolic blood pressure, diastolic blood pressure, renal biopsy, NS type, relapse rate before RTX, relapse rate after RTX, and previous treatments), and clinical data (RTX first dose, RTX total dose, RTX infusion interval, and concomitant use of steroids and immunosuppressants [ISs]).

## 4. Treatment protocol

RTX was administered at 375 mg/m^2^ body surface area or 1000 mg with each infusion. Based on their laboratory results, the patients received RTX therapy intermittently. Patients who experienced disease recurrence were withdrawn from the study.

We evaluated all patients before RTX infusion to exclude patients who had contraindications. Routine blood parameters, electrolytes, cardiac enzymes, troponin, chest CT images and electrocardiogram (ECG) findings were collected prior to infusion. To minimize infusion reactions, methylprednisolone and diphenhydramine hydrochloride were given before drug infusion. ECG monitoring was performed during infusion.

## 5. Definitions

CR is defined as proteinuria <0.3 g/24 h in the absence of kidney function deterioration along with normal serum albumin.

Partial remission is defined as proteinuria <3.5 g/24 h and a urine protein reduction of at least 50% compared to baseline, with stable kidney function and albumin rising.

Recurrence is defined as 24 hours urine protein >3.5 g/d again after CR or partial remission.

Relapse frequency is defined as the number of recurrences over time, measured in years.

B-cell reconstitution is defined as absolute CD19-positive count >5 cells/μL through peripheral blood flow cytometry.

## 6. Statistical analysis

Statistical analysis was performed using Statistical Product and Service Solutions (SPSS) 23.0 (IBM Corp., Armonk). The normality of the distribution of quantitative variables was evaluated using the Shapiro–Wilk test. All continuous variables are expressed as the mean ± standard deviation or median (minimum–maximum). We used nonparametric tests and ANOVA for between-group comparisons. The Wilcoxon signed-rank test was used to compare the paired recurrence rates before and after RTX administration. Statistical analyses for factors associated with recurrence were performed using the one-sample *t*-test, Mann–Whitney *U* test (for independent nonparametric comparisons), and chi-square test. The chi-square test was applied to assess the correlation between recurrence and administration of additional medication of RTX every year. All *P* values were 2-sided. *P *< .05 was considered significant.

## 7. Results

### 7.1. Baseline characteristics

The baseline characteristics of the study population are summarized in Table 1. The median age at disease diagnosis was 18.3 years (10.2–47.5 years) and the median age at the start of RTX treatment was 21.7 years (15.3–48.5 years). Kidney biopsy revealed MCD without acute tubular necrosis in all patients. The baseline 24-hour urine protein level was 0.13 g (0.01–2.4 g). The serum ALB concentration was 42.08 ± 3.83 g/L. The serum creatinine level was 69.50 ± 12.57 µmol/L. The baseline estimated glomerular filtration rate was 123.01 ± 13.50 mL/min/1.73 m^2^. The CHOL concentration was 4.51 ± 0.90 mmol/L. The number of CD19+ B cells was 243 (106–642)/μL. The mean follow-up time was 31.1 ± 9.6 months.

**Table 1:** The baseline characteristics of the study population.

Characteristics	Patients of refractory MCD
Age years	
Age at disease diagnosis	18.3 (10.2–47.5)
Age at RTX start	21.7 (15.3–48.5)
Sex ratio (F/M)	3/11
Comorbidities	
Diabetes, n (%)	1 (7.1)
Thromboembolic disease, n (%)	1 (7.1)
Coronary heart disease	1 (7.1)
Blood-pressure, mm Hg, mean ± SD	
Systolic	121.7 ± 9.1
Diastolic	84.5 ± 9.5
Renal biopsy	
MCD, n (%)	14 (100)
MCD + ATN, n (%)	0 (0)
NS type	
SDNS, n (%)	8 (57.1)
FRNS, n (%)	4 (28.6)
SRNS, n (%)	2 (14.3)
Previous IS treatments	
Steroid, n (%)	13 (92.9)
Calcineurin inhibitor, n, (%)	8 (57.1)
Cyclophosphamide, n (%)	1 (7.1)
Leflunomide	2 (14.3)
Tripterygium glycosides	1 (7.1)
Mycophenolate mofetil	1 (7.1)
Three or more immunosuppressants, n (%)	4 (28.6)
Relapse times before RTX, times/year	1.87 (0.43–2.69)
24-h urinary protein excretion, g	0.13 (0.01–2.4)
Serum albumin, g/L	42.08 ± 3.83
Serum creatinine, µmol/L	69.50 ± 12.57
eGFR (CKD-EPI), mL/min × 1.73 m^2^	123.01 ± 13.50
CHOL, mmol/L	4.51 ± 0.90
CD19 positive B-cells, number/µL	243 (106–642)
Follow-up time, months	31.1 ± 9.6

Quantitative data were expressed as median (minimum–maximum) or mean ± standard deviation (SD) categorical data were presented as frequencies.

ATN = acute tubular necrosis, eGFR = estimated glomerular filtration rate, FRNS = frequently relapsing nephrotic syndrome, IS = immunosupressant, MCD = minimal change disease, NS = nephrotic syndrome, RTX = rituximab, SD = standard deviation, SDNS = steroid-dependent nephrotic syndrome, SRNS = steriod-resistant nephrotic syndrome.

### 7.2. RTX dose

RTX dosing regimens over time of patients are summarized in Table 2. RTX was administered at 375 mg/m^2^ body surface area or 1000 mg with each infusion. Eight patients (patient 1, patient 3, patient 4, patient 5, patient 7, patient 9, patient 11, and patient 14) received the same dose of RTX 1 month after the first infusion. The cumulative dose of RTX ranged from 600 to 2500 mg after 6 months. The cumulative dose of RTX at the 12th was 1200 to 2500 mg. Two patients experienced a relapse within the 6th month to the 12th month. One patient had a relapse 9 months after receiving a single 600 mg of RTX. The other patient relapsed 12 months after receiving a single 1000 mg dose of RTX. By the second year, most patients were taking additional RTX which was 500 to 1400 mg and 2 patients did not use RTX of which 1 patient had a relapse.

**Table 2:** Rituximab dosing regimens over time of patients.

Patient number	The first dose of RTX, mg	RTX infusion 1 mo after the first infusion	Cumulative dose of RTX at the 6th mo, mg	Cumulative dose of RTX at the 12th mo, mg	Dose at the second year of RTX, mg	Dose at the third year of RTX, mg	Having a relapse during RTX administer
1	1000	1000	2000	2500	500	¶	No
2	1000	No	1000	1800	800	¶	No
3	1000	1000	2000	2500	1000	1000	No
4‡	1000	1000	2000	2500	500	0§	Yes
5	1000	1000	2500	2500	500	¶	No
6*	600	No	600	600§			Yes
7	1000	1000	2000	2500	600	¶	No
8	1000	No	1000	1500	500	¶	No
9‡	600	600	2200	2200	1400	0§	Yes
10*	1000	No	1000	1000§			Yes
11	600	600	1200	1700	‖	¶	No
12‡	700	No	1500	1500	0	0§	Yes
13	600	No	600	1200	600	¶	No
14†	700	700	1400	1400	1400§		Yes

RTX = rituximab.

* Relapsed within the first year.

† Relapsed within the second year.

‡ Relapsed within the third year.

§ Pre-relapse dose.

‖ Follow-up time <2 yr.

¶ Follow-up time <3 yr.

### 7.3. RTX interval of infusion

Based on the patients^,^ laboratory results, the patients received RTX therapy intermittently. The mean duration of the second infusion was 7.9 ± 2.1 months, with the exception of 3 patients who experienced a relapse (patient 6, patient 10, patient 14). The 3 patients who experienced relapse had been off medication for 9, 12, and 22 months, respectively. The interval between the second and third infusions averaged 11.8 ± 2.8 months beside 2 patients, one of whom had a relapse and the other did not use a third drug. The relapsed patient experienced a 22-month drug-free phase and then had a recurrence.

### 7.4. Concomitant use of steroids and ISs

Twelve patients stopped steroids or immunosuppressants during RTX treatment. The remaining 2 patients had a reduction in them. Dadas are summarized in Table 3. The duration of discontinuation of steroids or immunosuppressants was 10.1 ± 6.0 months after RTX treatment.

**Table 3:** The steroids and immunosuppresants during RTX treatment.

Patient number	IS in use at RTX initiaton	IS in use after RTX	IS stopping time
1	MP 4	S	6
2	PSL 15 TAC 2	S	8
3	CSA 100	S	6
4	MP 6 TAC 2	S	12
5	PSL 5 CSA100	S	2
6	PSL 20	R	–
7	PSL 7.5	S	16
8	PSL20	S	13
9	PSL 35 TAC 3	S	24
10	TAC 2	R	–
11	PSL 20 CSA 150	S	10
12	MP 16 CSA150	S	4
13	PSL30 TAC 3	S	12
14	PSL 5 CSA 50	S	8

CSA = cyclosporin A, IS = immunosuppressant, MP = methylprednisolone, PSL = prednisolone, R = reduce the dose of immunosupressants, RTX = rituximab, S = stop the use of immunosupressants, TAC = tacrolimus.

### 7.5. CD19-positive B-cells

The CD19-positive B-cell count was measured to be 243 (106–642)/μL before RTX treatment. After first infusion of RTX, CD19-positive B cells were completely eradicated in all patients. We intermittently monitored CD19-positive B-cells of patients and found that B-cell reconstitution occurred approximately 6 months after the administration of RTX. CD19-positive B-cell count was not associated with recurrence.

### 7.6. The relapse rate and relapse-related factors

**Figure 1.**
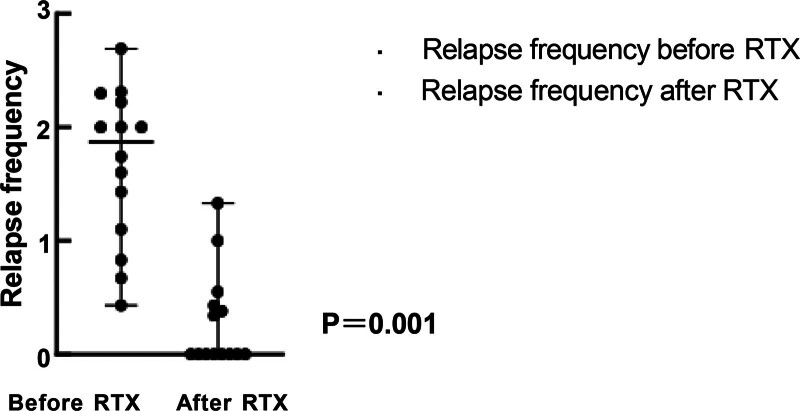
Relapse frequency before and after RTX therapy. Relapse frequency is defined as the number of recurrences over time, measured in years. This frequency is presented as the median number of relapses per year, with the range reported as minimum and maximum values. RTX = rituximab.

**Figure 2.**
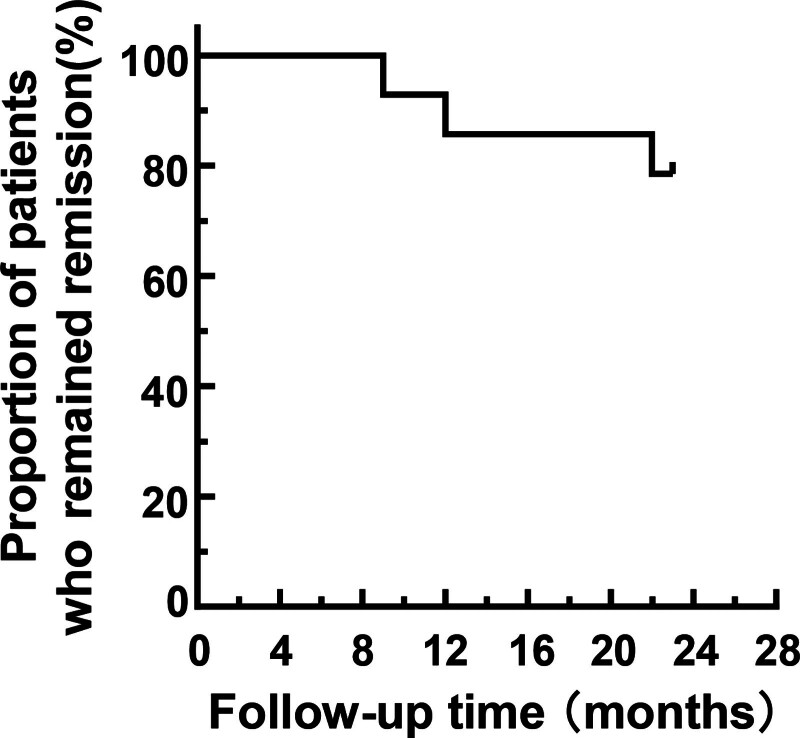
Kaplan–Meier curve for relapse-free survival in 22 mo. Kaplan–Meier survival analysis was performed to assess the relapse-free survival time.

Eight patients were in sustained remission, and the remaining 6 patients (patient 4, patient 6, patient 9, patient 10, patient 12 and patient 14) experienced a relapse. The relapse frequency significantly decreased from 1.87 (0.43, 2.69) to 0 (0.00, 1.33) before and after the application of RTX (*P* < .05; Fig. 1). The Kaplan–Meier curve for remission maintenance is shown in Figure 2.

Correlation analysis showed that recurrence was not related to the age at disease diagnosis, age of RTX application, sex, comorbidities, blood pressure, type of NS, immunosuppressive use, prerecurrence rate or cumulative dose of RTX (Table S1A, Supplemental Digital Content, https://links.lww.com/MD/R9). We performed a correlation analysis on patients who experienced recurrence in different years. The recurrence within the first year was associated with the cumulative dose at 6th month, the average annual dose of RTX and the administration of additional medication during the year. They all showed positive correlation. There was no clear correlation with the infusion interval (Table S1B, Supplemental Digital Content, https://links.lww.com/MD/R9). The recurrence within the second year was associated with the administration of additional medication of RTX during the second year (Table S1C, Supplemental Digital Content, https://links.lww.com/MD/R9).

### 7.7. Adverse events

Patient monitoring was conducted as follows: continuous vital signs and ECG during RTX infusion; education on self-reporting of adverse events post-discharge; and long-term, monthly laboratory assessments in an outpatient setting to screen for treatment-related effects. In our study, RTX was well tolerated. No serious adverse events were observed. Adverse events were predominantly mild or moderate and reversible. Three of the fourteen patients experienced transient skin rash on the body, which was resolved with a second dose of 40 mg of methylprednisolone and slowing the infusion rate. One of the fourteen patients experienced thoracic pain, which was relieved by slowing the infusion rate.

## 8. Discussion

MCD represents approximately 10% to 15% of patients with idiopathic NS in adults. Patients with refractory MCD, including steroid-dependent NS, steroid-resistant NS, and frequently relapsing NS, require long-term therapeutic strategies involving either prolonged corticosteroid maintenance therapy or combination regimens incorporating other immunosuppressive agents. Even so, recurrences often occur, thus accelerating disease progression and seriously affect normal life. An increasing number of studies have confirmed that RTX is a promising therapeutic option for MCD.^[3–5,8,9]^ The pathogenesis of MCD is undefined. Diffuse effacement of podocyte foot processes, the absence of electron-dense deposits or negative immunofluorescence staining are the characteristics of MCD.^[2]^ Dysregulated T cells can produce abnormally increased circulating permeability factors, thus driving the injury of podocytes in MCD. Different lines of evidence suggest a reduced function of regulatory T cells in MCD in adult patients. B lymphocytes play a crucial role in the pathogenesis of idiopathic MCD.^[10]^ RTX can eliminate B cells and disrupt B-cell–T-cell interactions, thus reducing the production of permeability factors. Additionally, RTX plays a role in B-cell-independent mechanisms. Perosa et al reported that RTX might cross-react with sphingomyelin-phosphodiesterase-acid-like-3b rather than acting directly on antibody production. RTX might prevent actin cytoskeleton remodeling in podocytes by preserving sphingolipid-related enzymes, sphingomyelin-phosphodiesterase-acid-like-3b and ASMase activity.^[11]^ The podocyte cytoskeleton may be a direct target for RTX through a B lymphocyte-independent mechanism.^[12]^ The recent discovery of nephrin autoantibodies in a subset with MCD provides further support for MCD etiology, which may lead to a new molecular classification of nephrin autoantibody MCD and instigate new therapeutics for MCD.^[13]^ Patients with anti-slit antibodies show a good response to second-line immunosuppressants and a favorable long-term outcomes.^[14]^ The detection of anti-slit antibodies in kidney biopsies, in addition to the measurement of anti-nephrin antibodies in the serum of patients with steroid-resistant NS, enables the identification of patients who are more likely to respond to intense immunosuppression. Our study did not investigate these factors or antibodies.

In the remission maintenance period of MCD, the regimens of RTX are uncertain. There are no treatment guidelines that specify infusion intervals, doses, or when the drug can be discontinued. Among relapsing ANCA-vasculitis patients, fixed-interval RTX retreatment has been suggested or considered a more prolonged period of remission.^[15]^ A few studies have provided suggestions for the induction period of MCD. It varies greatly from 500 mg, 375 mg/m^2^ or 1000 mg at 1 or 2 time points, even once weekly for 4 weeks.^[10,16]^ Takei et al performed a prospective trial in 25 MCNS patients (steroid-dependent minimal-change NS) who used a single dose of RTX (375 mg/m^2^ body surface area) twice at an interval of 6 months.^[16]^ Among the 13 patients with MCD who were in remission with prednisolone, IS treatment or both, most patients received RTX 2 to 4 times at 6-month interval with a single dose of 375 mg/m^2^ body surface area (maximum 500 mg).^[8]^ RTX was administered at 375 mg/m^2^ body surface area or 1000 mg every infusion, while some patients were remedicated with RTX 1 month after the first infusion in our study. In the previous short-term studies of RTX in MCD patients, the infusion interval in remission phase was mostly fixed at 6-month interval. In our research, the mean duration of the second infusion was 7.9 ± 2.1 months which represented an interval of infusion longer than 6 months for the majority of patients. The relapsed patients had been off medication for 9, 12, and 22 months, respectively. The interval between the second and third infusion averaged 11.8 ± 2.8 months which was longer than the last interval. Also, we found that patients who had the administration of additional medication during the year had a lower recurrence rate. We discovered that recurrence was associated with the administration of additional medication of RTX every year. Combined with patients infusion intervals of our study, we speculate that an additional dose of RTX around 6 months after initial treatment and annual additional infusions of RTX in subsequent treatments appear to be beneficial in reducing recurrence. Larger controlled studies are necessary to establish long-term efficacy. For the 3 patients who relapsed after an extended period without drug treatment (20–22 months between infusions), it appeared that complete cessation of treatment was not feasible at this time. This study does not provide an answer as to when the medication can be completely terminated due to time constraints. So far, no studies have been conducted on the infusion interval and time to complete drug withdrawal.

In terms of reducing the relapse rate or reducing the steroid dose, RTX greatly contributes. Takei et al confirmed that RTX therapy was associated with a reduction in relapse frequency and in the total dose of prednisolone needed. Munyentwali et al analyzed 17 patients with steroid-dependent or frequently relapsing minimal change NS. Some of them stopped or had a reduction in the use of steroid and IS drugs.^[17]^ Recently, a report verified that RTX treatment resulted in an 80.3% remission rate.^[4]^ In another systematic review and meta-analysis, RTX treatment resulted in a 91.6% CR rate, and 27.4% of patients experienced at least one relapse after RTX treatment. RTX is associated with trivial adverse events and good tolerance.^[5]^ RTX has the potential to maintain prolonged remission. In our study, the median relapse frequency significantly decreased from 1.87 (0.43–2.69) to 0 (0–1.33) times/yr, which was the same as that reported in some previous studies. Twelve patients stopped steroids or immunosuppressants and the remaining 2 patients experienced a reduction during RTX treatment. This finding reinforced the role of RTX as a glucocorticoid or IS-sparing agent. Early termination of immunosuppressants could greatly reduce harm to the body. RTX has also been shown to be effective in treating steroid-resistant NS. To minimize the side effects of conventional drug therapy, RTX therapy should be administered as early as possible during the remission period.

The relationship between relapse and CD19-positive B-cell repletion has been reported in many studies.^[3,16,17]^ Some studies have concluded that increase in CD19-positive B-cell is associated with disease relapse. However, Takei T concluded that B-cell restitution was not related to relapse.^[16]^ In our study, relapse had no connection with B-cell reconstitution. Current research suggests that many factors are associated with recurrence. The blood concentration of RTX affects disease function. A few studies have suggested that urinary loss of RTX shortens the B-cell depletion period, which leads to relapse.^[17,18]^ Unfortunately, RTX concentrations were not measured in our study. The type of disease, age, concomitant use of immunosuppressants, and dose and frequency of RTX are factors affecting the duration of B-cell depletion.^[19,20]^

Clinicians need to provide appropriate treatment plans for each patient’s condition to slow the deterioration of kidney function, prevent complications, and help patients return to normal life.

In summary, our findings corroborate existing evidence that RTX therapy effectively reduces relapse rates and diminishes the dependency on corticosteroids and other immunosuppressive agents in the studied cohort. Despite this established efficacy, a significant gap remains in the post-therapy guidelines regarding standardized maintenance protocols for sustaining remission. The present study, with its extended follow-up duration, provides a detailed analysis of maintenance strategies by evaluating RTX dosing, intervals, and predictors of relapse. These insights culminate in novel conclusions and evidence-based recommendations for maintaining long-term remission. However, the present study has several limitations. First, its retrospective, single-center design and the absence of a control or comparison group constrain the generalizability of the findings. Secondly, the lack of therapeutic drug monitoring for RTX, combined with heterogeneity in dosing regimens and different follow-up periods, may have influenced the interpretation of treatment outcomes.

## 9. Conclusions

RTX is associated with reduced relapse frequency and earlier discontinuation of corticosteroids and immunosuppressants in refractory MCD patients during remission maintenance. Additional annual RTX administration represents an effective strategy for decreasing disease recurrence. Our findings suggest potential benefit of early administration. While findings are promising, larger controlled studies are necessary to establish optimal dosing intervals and long-term efficacy.

## Acknowledgments

We gratefully acknowledge all the participants who contributed to this study.

## Author contributions

**Conceptualization:** Li Tian, Lingling Xing, Shaomei Li.

**Data curation:** Li Tian, Liying Wen, Xuzhi Liang.

**Methodology:** Li Tian, Ranjie Fu.

**Software:** Li Tian.

**Writing – original draft:** Li Tian.

**Writing – review & editing:** Li Tian, Shaomei Li.

## Supplementary Material


