# Supplementary material for: Rituximab for refractory minimal change disease: Long-term outcomes in the remission maintenance phase
Source: Medicine (Baltimore). 2025 Dec 26;104(52):e46798. doi: 10.1097/MD.0000000000046798 (PMC12746990; doi:10.1097/MD.0000000000046798)
Supplement: Supplementary file 1 [file medi-104-e46798-s001.docx]

| Characteristics | Relapse group | Non-relapse group | P |
| --- | --- | --- | --- |
| age at disease diagnosis | 18.3(11.0-36.5) | 17.1(10.2-47.5) | P＞0.05 |
| age at RTX start | 21.2(15.8-40) | 22.3(15.3-48.5) | P＞0.05 |
| Sex ratio (F/M) | 1/5 | 2/6 | P＞0.05 |
| NS type |  |  |  |
| SDNS, n (%) | 3(50%) | 5(62.5) | P＞0.05 |
| FRNS, n (%) | 2(33.3%) | 2(25%) | P＞0.05 |
| SRNS, n (%) | 1(16.7%) | 1(12.5%) | P＞0.05 |
| relapse times before RTX, times/year | 1.86±0.57 | 1.52±0.76 | P＞0.05 |
| cumulative dose of rituximab, mg | 1850±1183 | 2775±1062 | P＞0.05 |

**Supplemental file Table 1a. Factors not associated with relapse in univariate analysis**

NS, nephrotic syndrome; SDNS, steroid-dependent nephrotic syndrome; FRNS, frequently relapsing nephrotic syndrome; SRNS, steriod-resistant nephrotic syndrome; RTX, rituximab.

**Supplemental file Table 1b.** Patients in the first year of RTX therapy

| Characteristics | Twelve non-Relapse patients during the first year | Two relapse patients during the first year |
| --- | --- | --- |
| dose of RTX in the first month, mg | 1300 (600-2000) | 800 (600-1000) |
| *****cumulative dose at 6th month, mg | 1750 (600-2500) | 800 (600-1000) |
| *****average annual dose of RTX, mg | 2000 (1200-2500) | 900(800-1000) |
| *****addition of rituximab during the first year, % | 92% | 0% |
| The infusion interval between the first and second treatment, month | 9 (4-12) | 10.5(9-12) |

Quantitative data were expressed as median (minimum，maximum)

*****Statistically significant difference

RTX, rituximab.

| Characteristics | Ten non-Relapse patients during the first year | One relapse  patients during the first year |
| --- | --- | --- |
| cumulative dose at the first year, mg | 2350 (1200-2500) | 1400 |
| cumulative dose at the second year, mg | 3000 (1500-3600) | 1400 |
| Average annual dose of RTX, mg | 550(0-1400) | 0 |
| *****addition of rituximab during the second year, % | 90% | 0% |

**Supplemental file Table 1c.** Patients in the second year of RTX therapy

Quantitative data were expressed as median (minimum，maximum)

*****Statistically significant difference

RTX, rituximab.
